# Comprehensive circulating microRNA profile as a supersensitive biomarker for early-stage lung cancer screening

**DOI:** 10.1007/s00432-023-04728-9

**Published:** 2023-04-19

**Authors:** Masayasu Inagaki, Makoto Uchiyama, Kanae Yoshikawa-Kawabe, Masafumi Ito, Hideki Murakami, Masaharu Gunji, Makoto Minoshima, Takashi Kohnoh, Ryota Ito, Yuta Kodama, Mari Tanaka-Sakai, Atsushi Nakase, Nozomi Goto, Yusuke Tsushima, Shoich Mori, Masahiro Kozuka, Ryo Otomo, Mitsuharu Hirai, Masahiko Fujino, Toshihiko Yokoyama

**Affiliations:** 1 Department of Respiratory Medicine, Japanese Red Cross Aichi Medical Center Nagoya Daiichi Hospital, 3-35 Michishita-Cho, Nakamura-Ku, Nagoya, Aichi 453-8511 Japan; 2 Research and Development Division, ARKRAY, Inc., Yousuien-Nai, 59 Gansuin-Cho, Kamigyo-Ku, Kyoto, 602-0008 Japan; 3 Department of Pathology, Japanese Red Cross Aichi Medical Center Nagoya Daiichi Hospital, Nagoya, Aichi 453-8511 Japan; 4 Department of Cytology and Molecular Pathology, Japanese Red Cross Aichi Medical Center Nagoya Daiichi Hospital, Nagoya, Aichi 453-8511 Japan; 5 Department of Respiratory Surgery, Japanese Red Cross Aichi Medical Center Nagoya Daiichi Hospital, Nagoya, Aichi 453-8511 Japan

**Keywords:** Lung cancer, microRNA, Serum, Next-generation sequencing, Automated machine learning

## Abstract

**Purpose:**

Less-invasive early diagnosis of lung cancer is essential for improving patient survival rates. The purpose of this study is to demonstrate that serum comprehensive miRNA profile is high sensitive biomarker to early-stage lung cancer in direct comparison to the conventional blood biomarker using next-generation sequencing (NGS) technology combined with automated machine learning (AutoML).

**Methods:**

We first evaluated the reproducibility of our measurement system using Pearson’s correlation coefficients between samples derived from a single pooled RNA sample. To generate comprehensive miRNA profile, we performed NGS analysis of miRNAs in 262 serum samples. Among the discovery set (57 patients with lung cancer and 57 healthy controls), 1123 miRNA-based diagnostic models for lung cancer detection were constructed and screened using AutoML technology. The diagnostic faculty of the best performance model was evaluated by inspecting the validation samples (74 patients with lung cancer and 74 healthy controls).

**Results:**

The Pearson’s correlation coefficients between samples derived from the pooled RNA sample ≥ 0.98. In the validation analysis, the best model showed a high AUC score (0.98) and a high sensitivity for early stage lung cancer (85.7%, *n* = 28). Furthermore, in comparison to carcinoembryonic antigen (CEA), a conventional blood biomarker for adenocarcinoma, the miRNA-based model showed higher sensitivity for early-stage lung adenocarcinoma (CEA, 27.8%, *n* = 18; miRNA-based model, 77.8%, *n* = 18).

**Conclusion:**

The miRNA-based diagnostic model showed a high sensitivity for lung cancer, including early-stage disease. Our study provides the experimental evidence that serum comprehensive miRNA profile can be a highly sensitive blood biomarker for early-stage lung cancer.

**Supplementary Information:**

The online version contains supplementary material available at 10.1007/s00432-023-04728-9.

## Introduction

Lung cancer is the second most common malignancy and the first leading cause of cancer-related deaths in the world (Sung et al. 2021). Since the 5-year survival rate for patients with lung cancer patients diagnosed at an early stage is approximately 70% or more, which drops to less than 10% for patients diagnosed at an advanced stage, prompt detection might be a critical step in influencing the prognosis of lung cancer (Goldstraw et al. 2016). Although low-dose CT has been considered a screening tool for detecting lung cancer, the high false-positive rates of this technology might lead to over-diagnosis and consequently over-treatment (Bach et al. 2012). Hence, the field of early detection of lung cancer remains a challenge.

MicroRNAs(miRNAs), a class of small single-stranded noncoding RNAs (Hermeking 2012), play important roles in various cellular processes, such as cell differentiation and apoptosis, by regulating gene expression (O'Brien et al. 2018). Several recent studies have reported that the expression of various miRNAs changes dynamically in the body fluids of patients with lung cancer (Sozzi et al. 2014; Jin et al. 2017; Asakura et al. 2020).

Next-generation sequencing (NGS) ensures high accuracy in distinguishing miRNAs at a single-base resolution, and is suitable for generating comprehensive miRNA profiles. NGS presents a high detection sensitivity and a high level of technical reproducibility compared to that of other technologies (Tam et al. 2014). However, no previous study has demonstrated the clinical utility of a lung cancer discrimination model constructed using comprehensive miRNA profiles generated via NGS-based measurements.

Machine learning (ML) is one of the most powerful tools in the biomedical classification analysis of various diseases including coronavirus disease 2019 (COVID-19), neurological disorders and cancer (Gao et al. 2020; Boutet et al. 2021; Kourou et al. 2014). However, constructing an ML model manually requires statistical knowledge and immense effort in significant coding, resulting in a difficulty of the wide distribution of this powerful tool in biomedical research fields (Papoutsoglou et al. 2021).

Therefore, this study aimed to validate the ability of serum miRNA profiles to distinguish between patients with lung cancer and healthy controls by employing NGS technology in combination with automated machine learning (AutoML).

## Materials and methods

### Patient recruitment and sample collection

Serum was collected from patients with lung cancer at the Japanese Red Cross Aichi Medical Center Nagoya Daiichi Hospital (JRCN). All lung cancer cases were pathologically diagnosed using surgery or biopsy specimens and confirmed to be free of other malignancies at the time of blood sampling. The time interval between blood sampling and serum freezing at – 80 °C was observed strictly within the same day, and serum samples showing hemolysis were excluded. Patients with lung cancer were divided into a discovery dataset and a validation dataset according to their recruitment period (discovery dataset, from February 2021 to July 2021; validation dataset, from August 2021 to June 2022).

Serum was collected from healthy participants at the OCROM clinic (OCROMC), Osaka Pharmacology Clinical Research Hospital (OPHACH) and ToCROM clinic (TOCROMC). Inclusion criterion for healthy control participants were no history of malignant tumor according to self-reported medical history at the time of blood sampling and 1 year later. In our discovery dataset, 57 healthy serum samples were selected so that the age distribution matched that of participants with the lung cancer. In addition, in our validation dataset, 74 healthy serum samples were selected so that the age and sex distributions matched those of the lung cancer participants.

All individuals gave their written consent for the use of their serum samples and clinical information. This study was reviewed and approved by the Research Ethics Committee of JRCN (Registry number: 2020-107), OCROMC (Registry number: 1108), OPHACH (Registry number: 1108), TOCROMC (Registry number: 1108) and ARKRAY, Inc. (Registry number: EC590016).

### Blood sample collection and miRNA extraction from serum

All blood samples were collected in serum-separating tubes. After blood collection, these sera were separated by centrifugation, and aliquoted into cryotubes. Then, these sera were frozen at − 80 °C until miRNA extraction. RNA samples containing miRNA were extracted from the serum using the Maxwell® RSC miRNA Plasma and Serum kit (Promega). For monitoring RNA extraction, QIAseq miRNA Library QC Spike-ins (Qiagen) were spiked into each serum samples. miRNA concentrations were quantified using Qubit™ microRNA Assay Kits (Thermo Fisher Scientific). These RNA samples were stored at − 80 °C until NGS library preparation.

### NGS library preparation and NGS

miRNA libraries were constructed using a QIAseq miRNA Library Kit (Qiagen) and the QIAseq miRNA NGS 96 Index IL (96) (Qiagen) with Agilent Bravo NGS (Agilent Technologies). The library size distribution was checked using a TapeStation HS D1000 system (Agilent Technologies). The library samples were pooled, and then the DNA solution, including PhiX control library (Illumina), was spiked in the sample mixture, consistent with the manufacture’s recommendations. The pooled sample was sequenced on four lanes of a NextSeq 500/550 High Output Kit (75 cycles) sequenced using a NextSeq 550 platform (Illumina). The reads were annotated using the QIAseq miRNA primary analysis pipeline provided by the GeneGlobe Data Analysis Center (https://geneglobe.qiagen.com/jp/analyze/). The sequencing output was mapped to miRBase v21 using the QIAseq miRNA Primary Analysis Pipeline.

### miRNA data normalization and production

Raw read counts were normalized using reads per million (RPM) in each of the samples, and then the normalized data were log2-transformed (Campbell et al. 2015). The offset did not include miRNAs with near-zero read counts in the discovery dataset because it was filtered to include only miRNAs with minimum read count values ≥ 15 reads in each of the profiled samples. The remaining 181 miRNAs were used for subsequent analyses and are shown in Table S1. In Table S1, the miRNAs were sorted in descending order based on their average RPM values among the samples in discovery dataset.

### Reproducibility verification of the reproducibility of comprehensive miRNA NGS analysis

**Fig. 1:**
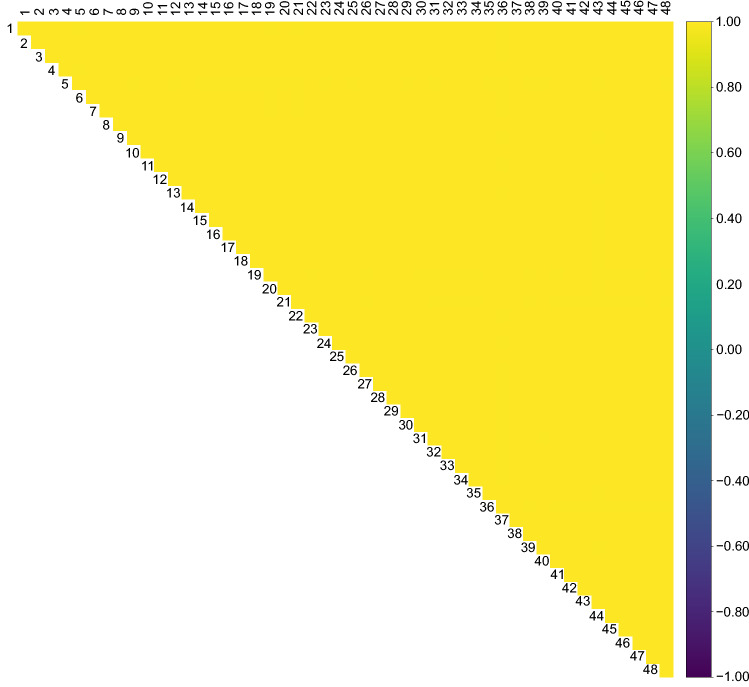
Pairwise correlation analysis of miRNA expressions between 48 samples derived from a single pooled RNA. All the sample data were acquired through a single NGS measurement. Each sample is assigned a sample number, and the correlation coefficients between samples are color coded in matrices

**Fig. 2:**
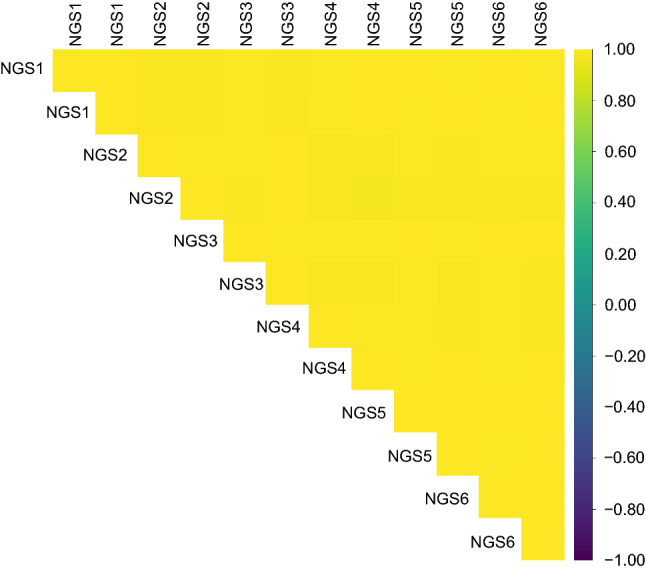
Pairwise correlation analysis of miRNA expressions between 12 samples derived from a single pooled RNA. Each measurement included two samples. Six measurements were performed in total (first measurement, NGS1; second measurement, NGS2; third measurement, NGS3; fourth measurement, NGS4; fifth measurement, NGS5; and sixth measurement, NGS6). The correlation coefficients between samples are color coded in matrices

We conducted pairwise correlation analysis of miRNAs to verify the reproducibility to determine comprehensive miRNA profile (Figs. 1 and 2). In Fig. 1, we analyzed miRNA expressions between 48 samples derived from a single pooled RNA. All the sample data were acquired through a single NGS measurement. In Fig. 2, we analyzed miRNA expressions between 12 samples derived from a single pooled RNA. Each measurement included two samples, and six measurements were performed in total. The single pooled RNA samples were derived from blood donated by the employees of ARKRAY, Inc., and the miRNAs analyzed are shown in Table S1.

### Screening of the cancer discrimination model using AutoML

To construct and screen cancer diagnostic models, we used the automated classification algorithm h2o.automl in the Java application H2O Flow of version 3.34.0.1, which was downloaded from https://www.h2o.ai/ (LeDell and Poirier 2020). To construct models, default random forest, extremely randomized forest, gradient boosting machine (GBM), and eXtream gradient boosting were used. A total of 181 miRNAs were used to construct miRNA-based diagnostic models (Table S1). The constructed models were ranked using a five-fold cross-validation area under the curve (AUC) score calculated by the H2O application in the discovery dataset to estimate the diagnostic performance without sacrificing a validation split. For further analysis, we then selected the primary model that exhibited the best five-fold cross-validation AUC score. Finally, the performance of the miRNA-based diagnostic model that was selected as the best model was evaluated using the validation dataset (Fig. 4). The highest F1 score computed by the H2O application in the five-fold cross-validation was used as the threshold of the best diagnostic model in the validation analysis.

### Tumor marker assays

Serum carcinoembryonic antigen (CEA) levels were determined using Alinity CEA Reagent Kit (Abbott Laboratories), whereas cytokeratin 19 fragment 21-1 (CYFRA21-1) levels were determined using Alinity CYFRA 21-1 Reagent Kit (Abbott Laboratories). Their reference range was respectively 0 to 5 ng/ml for CEA and 0 to 3.5 ng/ml for CYFRA 21-1.

### Statistical analysis

Pairwise correlation analysis, principal component analysis, box plots, and AUC calculation were conducted using the statistical analysis software R (version 4.0.3) and H2O Flow. Student’s *t* test for continuous variables and Fisher's exact test for categorical variables were used to analyze patient characteristics and diagnostic performance. To evaluate model performance (sensitivity and specificity), 95% confidence intervals (CIs) were calculated using the Wilson score method.

## Results

### Characteristics of the subjects

To generate miRNA expression profiles using NGS, 131 lung cancer serum samples from the JRCN and 131 healthy samples from the OPHACH, OCROMC, and ToCROMC were subjected to comprehensive NGS analysis. Lung cancer samples were divided into discovery (recruited from February 2021 to July 2021) and validation (recruited from August 2021 to June 2022) datasets. In both these datasets, healthy participants were selected so that the age distribution matched that of patients with lung cancer. The discovery dataset included 57 lung cancer samples and 57 healthy samples, and the validation dataset included 74 lung cancer samples and 74 healthy samples. The detailed patient characteristics for both datasets are shown in Table 1. In the validation dataset, we observed no significant differences in age and sex; however, the proportion of smokers was significantly higher among patients with lung cancer than among healthy participants (Fisher's exact test, *P* < 0.001).

**Table 1:** Clinical characteristics of lung cancer and healthy participants in the discovery and validation datasets

Characteristics	Discovery set (*N* = 114)		Validation set (*N* = 148)		
	Lung cancer (*N* = 57)	Healthy (*N* = 57)	Lung cancer (*N* = 74)	Healthy (*N* = 74)	*P*-value
Age, years mean(SD)	72.1 (8.8)	70.0 (9.9)	73.9 (8.5)	71.4 (9.9)	0.18^a^
Sex, *n* (%)					1^b^
Men	45 (78.9)	28 (49.1)	47 (63.5)	47 (63.5)	
Women	12 (21.1)	29 (50.9)	27 (36.5)	27 (36.5)	
Smoking status, *n* (%)					3.6 × 10^–4 b^
Current or Former	45 (78.9)	17 (29.8)	56 (75.7)	34 (45.9)	
Never	10 (17.5)	40 (70.2)	18 (24.3)	40 (54.1)	
NA	2 (3.5)	–	–	–	
Subtype, *n* (%)					
NSCLC	52 (91.2)	–	67 (90.5)	–	
AC	35 (61.4)	–	37 (50.0)	–	
SCC	11 (19.3)	–	21 (28.4)	–	
Others	6 (10.5)	–	9 (12.2)	–	
SCLC	5 (8.8)	–	7 (9.5)	–	
Stage, *n* (%)					
0/I	29 (50.9)	–	28 (37.8)	–	
II	4 (7.0)	–	7 (9.5)	–	
III	9 (15.8)	–	17 (23.0)	–	
IV	15 (26.3)	–	22 (29.7)	–	

*NSCLC* non-small cell lung carcinoma, *AC* adenocarcinoma, *SCC* squamous cell carcinoma, *Others* other non-small cell carcinoma, *SCLC* small cell lung carcinoma

^a^Student’s *t* test

^b^Fisher's exact test

### Technical validation of comprehensive NGS analysis of miRNA

First, we evaluated the reproducibility of our analytical system in identifying sample miRNA profiles by analyzing healthy samples from six different NGS measurements. Each measurement (NGS1, NGS2, NGS3, NGS4, NGS5, and NGS6) showed high percentages of reads mapping to miRNAs in the total reads, with an above averaging 30% (Fig. S1A). To explore NGS measurement biases, we performed principal component analysis (PCA) and boxplot analysis (BA) for each miRNA profile among healthy samples. The PCA and BA results showed no noticeable differences among NGS measurements (Fig. S1B, C). In addition, we evaluated the measurement reproducibility by analyzing the Pearson’s correlation coefficients of the miRNA profiles among 48 samples derived from a single pooled RNA sample in one NGS measurement. The results showed that all correlation coefficients were ≥ 0.99 (Fig. 1). Furthermore, pairwise correlation analysis showed remarkably high correlation coefficients ranging from 0.98 to 1.00 among 12 samples, which were derived from a single pooled RNA and prepared for six different NGS measurements (Fig. 2). These results demonstrated that our constructed system had a high reproducibility in identifying serum miRNA profiles.

### Screening of miRNA-based diagnosis models to select a best model for detecting lung cancer with high accuracy

**Fig. 3:**
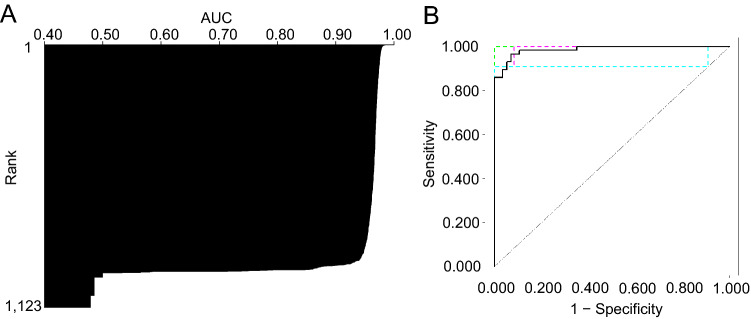
Screening of miRNA-based diagnostic models for detecting lung cancer. **A** Results of screening of miRNA-based diagnostic models for detecting lung cancer. AUC indicates the five-fold cross-validation AUC score computed by H2O. **B** ROC curves of a best miRNA-based diagnostic model for detecting lung cancer patient using comprehensive miRNA profile in five-fold cross-validation (combined ROC, black; ROC fold 1, green; ROC fold 2, red; ROC fold 3, purple; ROC fold 4, orange; and ROC fold 5, pink)

AutoML enables the construction of ML models without requiring immense time for coding and manually tuning hyper-parameters (Papoutsoglou et al. 2021). Among the discovery set (lung cancer, *n* = 57; healthy, *n* = 57), 1123 miRNA-based diagnostic models for lung cancer detection were constructed and screened using AutoML technology. A total of 181 miRNAs were used to construct the diagnostic models (Table S1). To select the best miRNA-based diagnostic model for the accurate detection of lung cancer, all 1123 models were ranked based on their five-fold cross-validation AUC scores in the discovery dataset (Fig. 3A). Finally, we selected the best five-fold cross-validation AUC score model, based on the GBM-algorithm, for further analysis. This miRNA-based diagnostic model exhibited a five-fold cross-validation AUC score (0.99) in the discovery dataset (Fig. 3B), suggesting that this model has high diagnostic performance.

### Evaluation of the diagnostic faculty of the selected miRNA-based diagnostic model for lung cancer

**Fig. 4:**
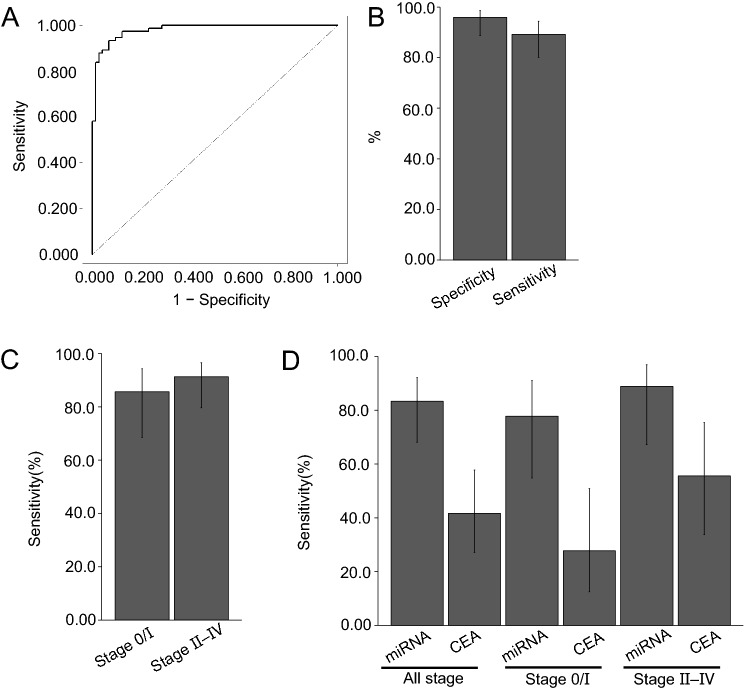
Evaluation of the performance of the best miRNA-based diagnostic model for detecting lung cancer patients in a validation dataset. **A** ROC curve of the miRNA-based diagnostic model in the validation dataset. **B** Sensitivity and specificity of the model. The bars represent 95% CIs. Two-sided Wilson CIs were calculated. **C** Sensitivity by cancer stage in the miRNA-based diagnostic model. The bars represent 95% CIs. Two-sided Wilson CIs were calculated. The threshold was the highest *F*1 score computed by H2O in the five-fold cross-validation. **D** Comparison of sensitivity to early-stage lung adenocarcinoma between the miRNA-based diagnostic model (miRNA) and CEA. The sensitivity of miRNA and CEA was assessed against lung adenocarcinoma samples for which CEA measurements were available [Total, 36 samples; Stage I, 18 samples; Stages II–IV, 18 samples]. The threshold of CEA is 5 ng/mL. Bars indicates 95% CIs. Two-sided Wilson CIs were calculated. The threshold of the miRNA-based diagnostic model was the highest *F*1 score computed by H_2_O in the five-fold cross-validation

We evaluated the performance of the selected miRNA-based diagnostic model for detecting lung cancer by applying it without further modification to a validation dataset (lung cancer, *n* = 74; healthy, *n* = 74). This model exhibited an AUC value of the 0.98 in the validation dataset with a high sensitivity [89.2% (95% CI 80.1–94.4%; *n* = 74)] and specificity [95.9% (95% CI 88.7–98.6%; *n* = 74)] (Fig. 4A, B). We also statistically analyzed the relation between prediction result of the miRNA-based model and smoking history. Our diagnostic model exhibited high lung cancer diagnostic performance in both smoking [Sensitivity, 91.1% (95% CI 80.7–96.1%; *n* = 56); Specificity, 94.1% (95% CI 80.9–98.3%; *n* = 34)] and non-smoking groups [Sensitivity, 83.3% (95% CI 60.8–94.2%; *n* = 28); Specificity, 97.5% (95% CI 87.1–99.6%; *n* = 40)] (Fig. S2).

Furthermore, we investigated the diagnostic ability of this model based on stage and histological subtype of lung cancer. Our miRNA-based diagnostic model also detected early-stage lung cancer (Stage 0/I) with high sensitivity [Stage 0/I, 85.7% (95% CI 68.5–94.3%; *n* = 28); Stages II–IV, 91.3% (95% CI: 79.7–96.6%; n = 46)] (Fig. 4C). Furthermore, it exhibited a high sensitivity, irrespective of the histological subtype. The sensitivity by each subtype is as follows: non-small cell lung carcinoma (NSCLC), 88.1% (95% CI: 78.2–93.8%; *n* = 67); adenocarcinoma (AC), 83.8% (95% CI 68.9–92.3%; *n* = 37); squamous cell carcinoma (SCC), 95.2% (95% CI 77.3–99.2%; *n* = 21); other non-small cell carcinoma (Others), 88.9% (95% CI 56.5–98.0%; *n* = 9); and small cell lung carcinoma(SCLC), 100.0% (95% CI 64.6–100.0%; *n* = 7) (Fig. S3). We also analyzed the sensitivity of two other well-known biomarkers of lung cancer, CEA and CYFRA21-1, corresponding to AC and SCC, respectively. Compared to CEA and CYFRA21-1, our model detected AC and SCC with higher sensitivity [(CEA, 41.7% (95% CI 27.1–57.8%; n = 36); CYFRA21-1, 57.1% (95% CI 36.5–75.5%; *n* = 21)] (Figs. 4D and S4). In particular, for early-stage AC, the sensitivity of this model was remarkably higher than that of CEA [miRNA, 77.8% (95% CI 54.8–91.0%; *n* = 18); CEA, 27.8% (95% CI 12.5–50.9%; *n* = 18)] (Fig. 4D). These results suggest that miRNA-based diagnostic models can detect lung cancer, including early-stage tumors, accurately.

## Discussion

CT scan is essential for the screening and diagnosing lung cancer. The National Lung Screening Trial showed that low-dose CT screening reduces lung cancer mortality among high-risk participants by 20% (National Lung Screening Trial Research Team 2011). In contrast, low-dose CT screening exhibits a high false-positive rate, possibly resulting in unnecessary examinations, such as invasive biopsies (Bach et al. 2012). Although collecting blood specimens only requires minimally invasive procedures and space occupancy without any radiation exposure, conventional serum biomarkers, such as CEA and CYFRA21-1, are not optimal especially for detecting early-stage cancer because of their low sensitivity and cancer stage-dependent differences (Rastel et al. 1994; Pujol et al. 1996; Bombardieri et al. 1994; Urabe et al. 2020; Molina et al. 1994). Thus, additional biomarkers for the early detection of lung cancer are strongly desirable in clinical practice. In the field of lung cancer research, two previous studies reported that several miRNAs in the blood, selected via comprehensive analysis of miRNA, showed high sensitivity for discriminating lung cancer, suggesting their efficacy as biomarkers (Jin et al. 2017; Asakura et al. 2020). However, numerous studies have revealed that each miRNA directly or indirectly controls the expression of various genes (Hermeking 2012), indicating the complexity of miRNA-mediated gene regulation in various diseases. Therefore, we focused on the possibility of comprehensive analysis using whole information of miRNAs without selection. NGS is a revolutionary technology for the simultaneous and accurate quantification of miRNAs, thereby enabling high-throughput comprehensive analysis of miRNA expressions (Tam et al. 2013). Furthermore, AutoML technology provides scientists with powerful ML-based insights without significant coding knowledge, time and effort (Papoutsoglou et al. 2021). In this study, we combined the comprehensive NGS analysis and AutoML technology to develop an miRNA-based diagnostic model for detecting lung cancer without the immense effort of programming and technical bias. Using the discovery set, AutoML constructed 1123 models, screened them and finally proposed the model with the outstanding performance (Figs. 3, 4). The sensitivity of our miRNA-based diagnostic model was higher than that of CEA and CYFRA21-1 for AC and SCC, respectively (Figs. 4D, S4). Furthermore, it should be noted that our model exhibited higher sensitivity than CEA for detecting early-stage lung AC (Fig. 4D). Our results suggest that the miRNA-based diagnostic model is more sensitive than the conventional cancer biomarkers in blood specimens. Interestingly, Sozzi et al. previously reported that a combination of low-dose CT and a miRNA discrimination model decreased the high false-positive rate of low-dose CT by 19.4–3.7% (Sozzi et al. 2014). Thus, a miRNA-based diagnosis combined with existing imaging examinations, can potentially improve diagnostic accuracy for early-stage lung cancer without unnecessary invasive procedures.

There are significant hurdles to overcome before the miRNA profile for cancer diagnosis can be used in clinical practice. When generating a comprehensive miRNA profile at the research level, NGS measurement is cost-effective and offers high-throughput compared to reverse transcription–quantitative PCR. However, compared to existing diagnostic methods, NGS is much more expensive for routine clinical use. Therefore, development of a cost-effective analytical method for miRNA-based diagnosis remains an issue warranting further study. However, considering the patient as a whole, a comprehensive miRNA profile may be versatile as NGS analysis can provide almost all the information on miRNA expression for each patient at the time of blood sampling. Numerous studies have reported that miRNA profiles enabled the detection of various diseases, such as other cancers and neurodegenerative diseases including Alzheimer’s disease (Jin et al. 2017; Asakura et al. 2020; Zhong et al. 2021; Zhu et al. 2017; Ying et al. 2020; Cheng et al. 2015; Suzuki et al. 2022; Geekiyanage et al. 2012). Hence, miRNA profiles can be available to perform other analyses of various disorders simultaneously, with minimally invasiveness.

One limitation of this study was the small sample size of healthy controls and patients with lung cancer due to the high cost of comprehensive NGS analysis. This limitation mainly affects the CIs of sensitivity to each histological subtype of lung cancer, making it difficult to interpret the diagnostic performance of this model (Figs. 4D, S2, S3, S4). Although the total number of comprehensive NGS analyses in this study was the three times larger than those in other previous study (Jin et al. 2017), further studies are needed to evaluate the capability of miRNA-based diagnostic models for each histological subtype of lung cancer. Another limitation of this study was difference in smoking history. Although our statistical analysis revealed that there was no significant relation between smoking history and lung cancer prediction in our validation dataset (Data not shown), smoking, especially, is well known as one of the major risk factor of lung cancer (Hecht. 1999). Moreover, in our validation dataset, CEA exhibited the considerably high sensitivity of about 30% for early-stage lung adenocarcinoma (Fig. 4D), suggesting that our validation dataset could be biased due to small sample size described above. Therefore, there remains a possibility that our datasets contain sample selection bias effecting on diagnostic performance.

In conclusion, using technologies of NGS and AutoML, we constructed a miRNA-based diagnostic model to detect early-stage lung cancer with high accuracy. Our results strongly support the clinical utility of serum miRNA profiles for lung cancer diagnosis, even in early-stage, encouraging further research toward clinical application.

## Supplementary Information

Below is the link to the electronic supplementary material.

Supplementary file1 (PDF 1904 KB)

## Data Availability

The datasets that support the findings of this study are available from the corresponding author upon reasonable request.
