# Supplementary material for: Comprehensive circulating microRNA profile as a supersensitive biomarker for early-stage lung cancer screening
Source: J Cancer Res Clin Oncol. 2023 Apr 19;149(11):8297–305. doi: 10.1007/s00432-023-04728-9 (PMC10115369; doi:10.1007/s00432-023-04728-9)
Supplement: Supplementary file 1 — Supplementary file1 (PDF 1904 KB) [file 432_2023_4728_MOESM1_ESM.pdf]

Supplementary Information

Comprehensive circulating microRNA profile as a supersensitive biomarker for early-stage lung cancer screening

Masayasu Inagaki, Makoto Uchiyama, Kanae Yoshikawa-Kawabe, Masafumi Ito, Hideki Murakami, Masaharu Gunji, Makoto Minoshima, Takashi Kohnoh, Ryota Ito, Yuta Kodama, Mari Tanaka-Sakai, Atsushi Nakase, Nozomi Goto, Yusuke Tsushima, Shoich Mori, Masahiro Kozuka, Ryo Otomo, Mitsuharu Hirai, Masahiko Fujino and Toshihiko Yokoyama

This PDF file includes:

Supplementary Fig. S1-S4

Supplementary Table S1

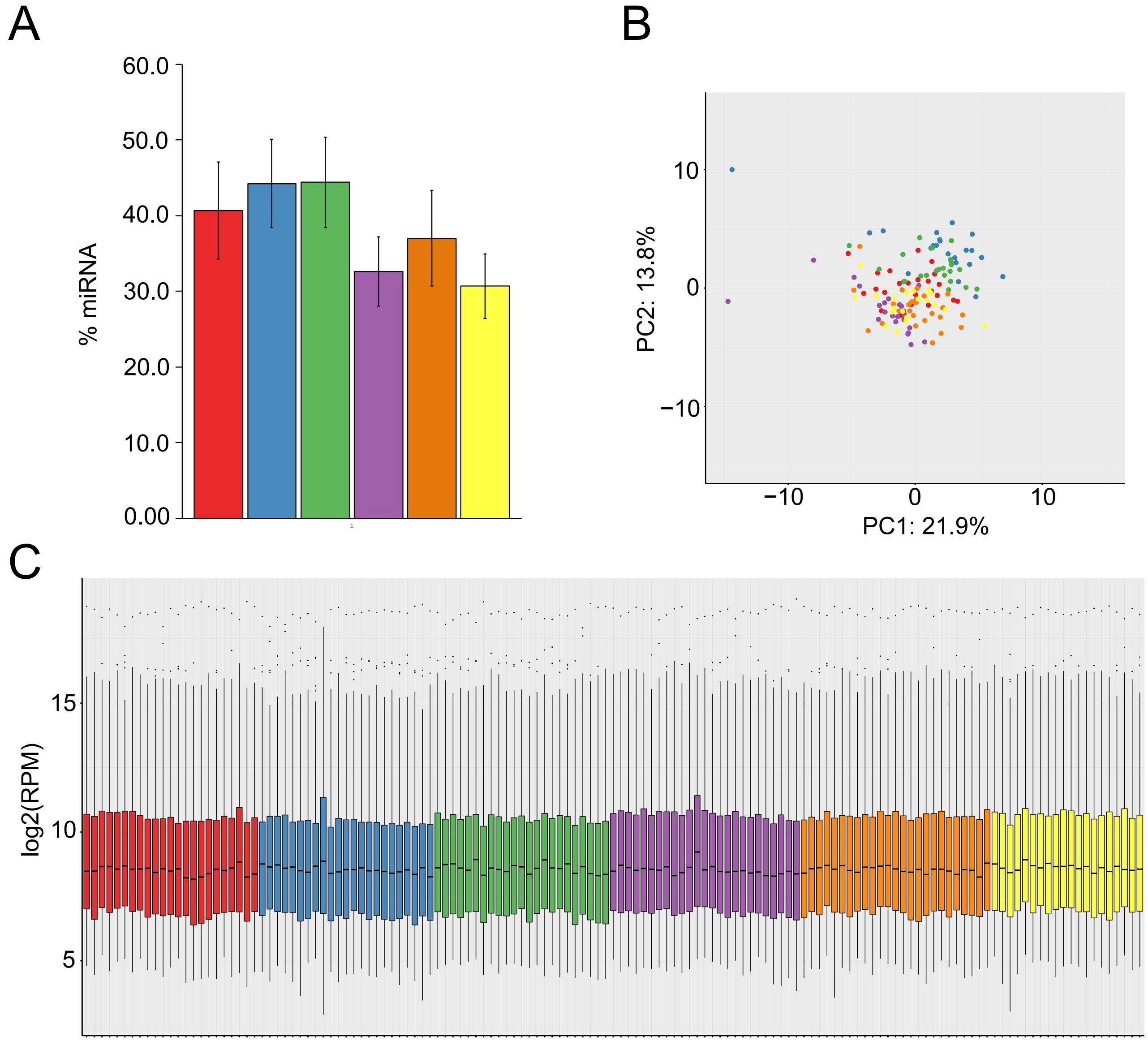

Fig. S1. Technical validation of NGS data acquisition. (A) Averaged percentages of miRNA raw reads to total reads in six different NGS library preparations (first measurement, NGS1, red; second measurement NGS2, blue; third measurement, NGS3,green; fourth measurement, NGS4, purple; fifth measurement, NGS5, orange; and sixth measurement, NGS6, yellow). (B) PCA plot for serum miRNA expression of healthy samples, colored by six different NGS measurements (first measurement, NGS1, red; second measurement, NGS2, blue; third measurement, NGS3, green; fourth measurement, NGS4, purple; fifth measurement, NGS5, orange; and sixth measurement, NGS6, yellow). Abbreviation: PC1, the first principal component; PC2, the second principal component. (C) Boxplots of log2-RPM value for each miRNA expression for each sample, colored by six different NGS measurements (first measurement, NGS1, red; second measurement, NGS2, blue; third measurement, NGS3, green; fourth measurement, NGS4, purple; fifth measurement, NGS5, orange;and sixth measurement, NGS6, yellow). In Fig. S1, all serums were collected in OCROMC, OPHACH and TOCROMC

.

Fig. S1

Inagaki et al.

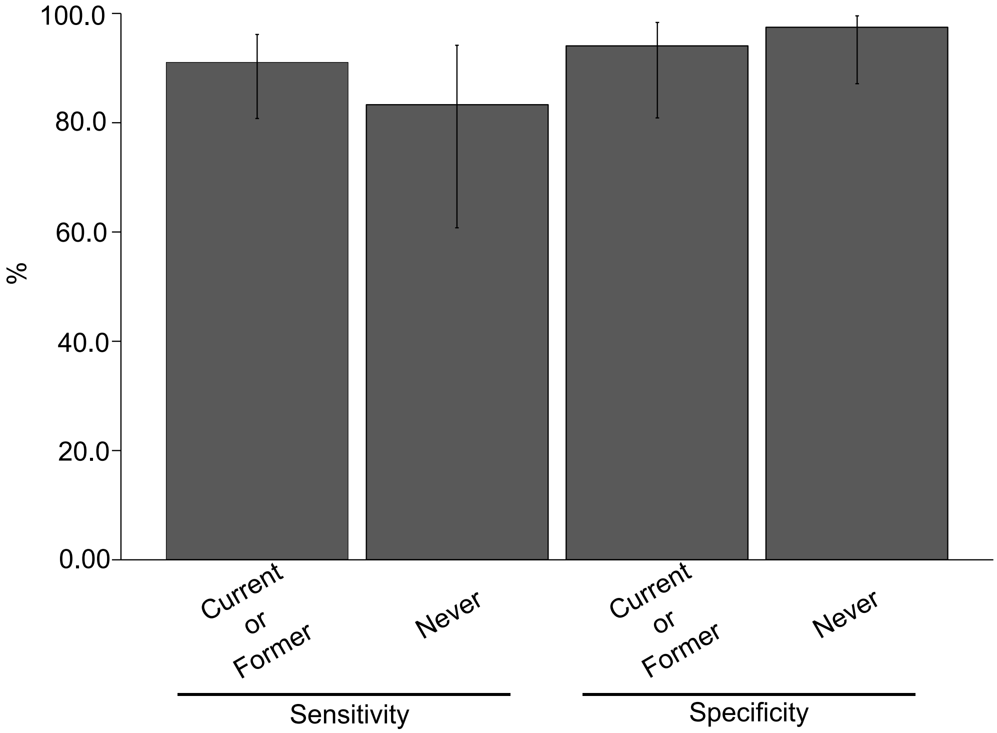

Fig. S2. Diagnostic performance of the miRNA-based diagnostic model in different smoking status in the validation set. The bars represent 95% CIs. Two-sided Wilson CIs were calculated. The threshold of the miRNA-based diagnostic model was the highest F1 score computed by H2O in the five-fold cross-validation. Abbreviation: Current, current smoker; Fomer, former smoker; Never, never smoking participant

Fig. S2
Inagaki et al.

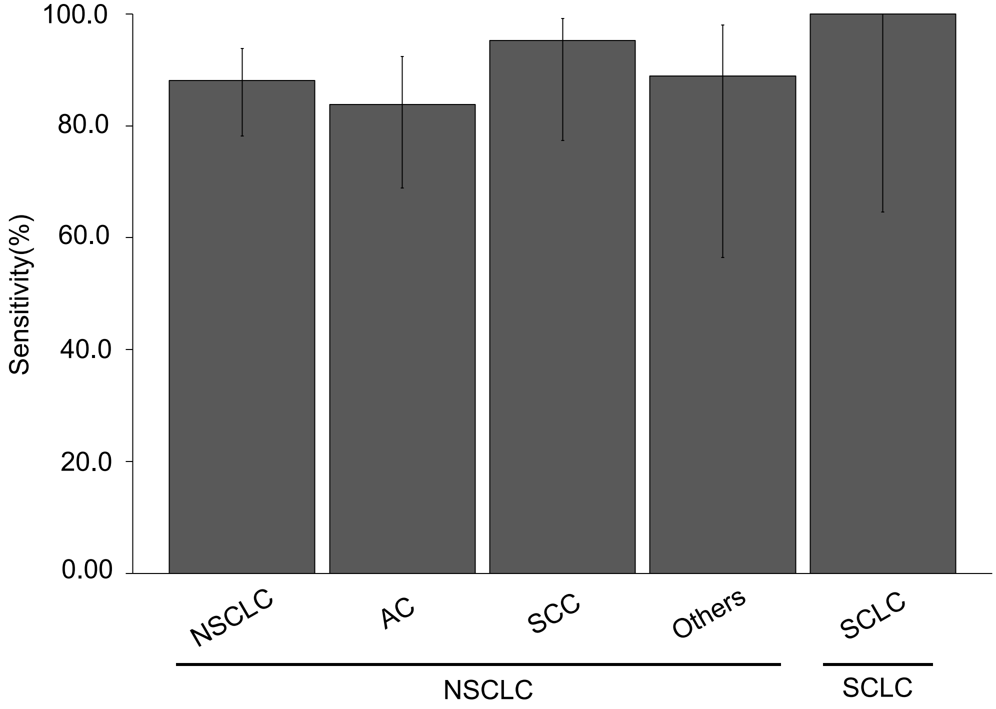

Fig. S3. Diagnostic performance of the miRNA-based diagnostic model in different histological types in the validation set. The bars represent 95% CIs. Two -sided Wilson CIs were calculated. The threshold of the miRNA-based diagnostic model was the highest F1 score computed by H2O in the five-fold cross-validation. Abbreviation: NSCLC, non-small cell lung carcinoma; AC, adenocarcinoma; SCC, squamous cell carcinoma; Others, non-small cell lung carcinoma; SCLC, small cell lung carcinoma.

Fig. S3
Inagaki et al.

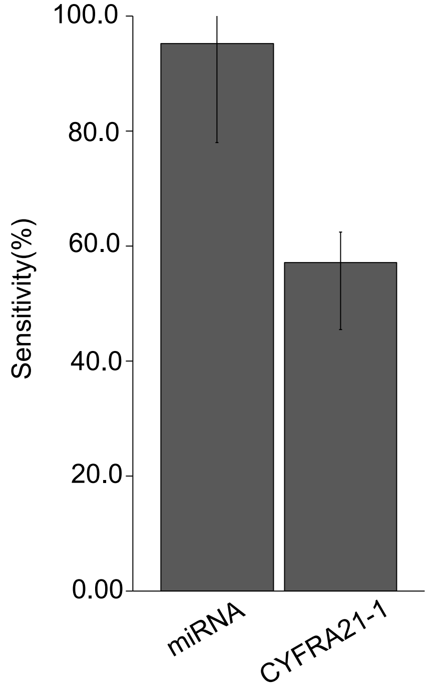

Fig. S4. Comparison of sensitivity to squamous cell carcinoma between the miRNA-based diagnostic model (miRNA) and CYFRA21-1. The threshold of CYFRA21-1 is 3.5 ng/mL. The bars represent 95% CIs. Two-sided Wilson CIs were calculated. The threshold of the miRNA-based diagnostic model was the highest F1 score computed by H2O in the five-fold cross-validation.

Fig. S4
Inagaki et al.

Table S1. List of analyzed miRNA

miRNA	Sequence
hsa-miR-16-5p	UAGCAGCACGUAAAUAUUGGCG
hsa-miR-223-3p	UGUCAGUUUGUCAAAUACCCCA
hsa-miR-126-3p	UCGUACCGUGAGUAAUAAUGCG
hsa-let-7a-5p	UGAGGUAGUAGGUUGUAUAGUU
hsa-let-7b-5p	UGAGGUAGUAGGUUGUGUGGUU
hsa-let-7f-5p	UGAGGUAGUAGAUUGUAUAGUU
hsa-let-7i-5p	UGAGGUAGUAGUUUGUGCUGUU
hsa-miR-122-5p	UGGAGUGUGACAAUGGUGUUUG
hsa-miR-486-5p	UCCUGUACUGAGCUGCCCCGAG
hsa-miR-101-3p	UACAGUACUGUGAUAACUGAA
hsa-miR-142-3p	UGUAGUGUUUCCUACUUUAUGGA
hsa-miR-26b-5p	UUCAAGUAAUUCAGGAUAGGU
hsa-miR-142-5p	CAUAAAGUAGAAAGCACUACU
hsa-miR-26a-5p	UUCAAGUAAUCCAGGAUAGGCU
hsa-miR-451a	AAACCGUUACCAUUACUGAGUU
hsa-miR-21-5p	UAGCUUAUCAGACUGAUGUUGA
hsa-miR-30e-5p	UGUAAACAUCCUUGACUGGAAG
hsa-miR-126-5p	CAUUAUUACUUUUGGUACGCG
hsa-miR-146a-5p	UGAGAACUGAAUUCCAUGGGUU
hsa-miR-93-5p	CAAAGUGCUGUUCGUGCAGGUAG
hsa-miR-25-3p	CAUUGCACUUGUCUCGGUCUGA
hsa-let-7g-5p	UGAGGUAGUAGUUUGUACAGUU
hsa-miR-30d-5p	UGUAAACAUCCCCGACUGGAAG
hsa-miR-199a-3p	ACAGUAGUCUGCACAUUGGUUA
hsa-miR-144-3p	UACAGUAUAGAUGAUGUACU
hsa-miR-103a-3p	AGCAGCAUUGUACAGGGCUAUGA
hsa-miR-92a-3p	UAUUGCACUUGUCCCGGCCUGU
hsa-miR-423-5p	UGAGGGGCAGAGAGCGAGACUUU

hsa-miR-191-5p	CAACGGAAUCCCAAAAGCAGCUG
hsa-miR-148a-3p	UCAGUGCACUACAGAACUUUGU
hsa-miR-185-5p	UGGAGAGAAAGGCAGUUCCUGA
hsa-miR-29c-3p	UAGCACCAUUUGAAAUCGGUUA
hsa-miR-23a-3p	AUCACAUUGCCAGGGAUUUCC
hsa-miR-143-3p	UGAGAUGAAGCACUGUAGCUC
hsa-miR-24-3p	UGGCUCAGUUCAGCAGGAACAG
hsa-miR-151a-3p	CUAGACUGAAGCUCCUUGAGG
hsa-miR-29a-3p	UAGCACCAUCUGAAAUCGGUUA
hsa-miR-150-5p	UCUCCCAACCCUUGUACCAGUG
hsa-miR-342-3p	UCUCACACAGAAAUCGCACCCGU
hsa-miR-27b-3p	UUCACAGUGGCUAAGUUCUGC
hsa-miR-148b-3p	UCAGUGCAUCACAGAACUUUGU
hsa-miR-192-5p	CUGACCUAUGAAUUGACAGCC
hsa-let-7d-5p	AGAGGUAGUAGGUUGCAUAGUU
hsa-miR-486-3p	CGGGGCAGCUCAGUACAGGAU
hsa-miR-20a-5p	UAAAGUGCUUAUAGUGCAGGUAG
hsa-miR-15b-5p	UAGCAGCACAUCAUGGUUUACA
hsa-miR-221-3p	AGCUACAUUGUCUGCUGGGUUUC
hsa-miR-27a-3p	UUCACAGUGGCUAAGUUCCGC
hsa-miR-15a-5p	UAGCAGCACAUAAUGGUUUGUG
hsa-miR-320a	AAAAGCUGGGUUGAGAGGGCGA
hsa-miR-140-3p	UACCACAGGGUAGAACCACGG
hsa-miR-125a-5p	UCCCUGAGACCCUUUAACCUGUGA
hsa-miR-19b-3p	UGUGCAAAUCCAUGCAAAACUGA
hsa-miR-425-5p	AAUGACACGAUCACUCCCGUUGA
hsa-miR-30a-5p	UGUAAACAUCCUCGACUGGAAG
hsa-miR-107	AGCAGCAUUGUACAGGGCUAUCA
hsa-miR-22-3p	AAGCUGCCAGUUGAAGAACUGU
hsa-miR-128-3p	UCACAGUGAACCGGUCUCUUU
hsa-miR-186-5p	CAAAGAAUUCUCCUUUUGGGCU
hsa-miR-335-5p	UCAAGAGCAAUAACGAAAAAUGU

hsa-miR-194-5p	UGUAACAGCAACUCCAUGUGGA
hsa-miR-182-5p	UUUGGCAAUGGUAGAACUCACACU
hsa-miR-29b-3p	UAGCACCAUUUGAAAUCAGUGUU
hsa-miR-340-5p	UUAUAAAGCAAUGAGACUGAUU
hsa-miR-125b-5p	UCCCUGAGACCCUAACUUGUGA
hsa-miR-223-5p	CGUGUAUUUGACAAGCUGAGUU
hsa-miR-103b	UCAUAGCCCUGUACAAUGCUGCU
hsa-let-7c-5p	UGAGGUAGUAGGUUGUAUGGUU
hsa-miR-199b-3p	ACAGUAGUCUGCACAUUGGUUA
hsa-miR-16-2-3p	CCAAUAUUACUGUGCUGCUUUA
hsa-miR-23b-3p	AUCACAUUGCCAGGGAUUACC
hsa-miR-1277-5p	AAAUAUAUAUAUAUAUGUACGUAU
hsa-miR-374a-5p	UUAUAAUACAACCUGAUAAGUG
hsa-miR-144-5p	GGAUAUCAUCAUAUACUGUAAG
hsa-miR-146b-5p	UGAGAACUGAAUUCCAUAGGCU
hsa-miR-195-5p	UAGCAGCACAGAAAUAUUGGC
hsa-miR-7-5p	UGGAAGACUAGUGAUUUUGUUGU
hsa-miR-19a-3p	UGUGCAAAUCUAUGCAAAACUGA
hsa-miR-28-3p	CACUAGAUUGUGAGCUCCUGGA
hsa-miR-190a-5p	UGAUAUGUUUGAUAUAUUAGGU
hsa-miR-584-5p	UUAUGGUUUGCCUGGGACUGAG
hsa-miR-30c-5p	UGUAAACAUCCUACACUCUCAGC
hsa-miR-98-5p	UGAGGUAGUAAGUUGUAUUGUU
hsa-miR-101-5p	CAGUUAUCACAGUGCUGAUGCU
hsa-let-7b-3p	CUAUACAACCUACUGCCUUCCC
hsa-miR-454-3p	UAGUGCAAUAUUGCUUAUAGGGU
hsa-miR-181a-5p	AACAUUCAACGCUGUCGGUGAGU
hsa-let-7e-5p	UGAGGUAGGAGGUUGUAUAGUU
hsa-miR-361-5p	UUAUCAGAAUCUCCAGGGGUAC
hsa-miR-155-5p	UUAAUGCUAAUCGUGAUAGGGGU
hsa-miR-744-5p	UGCGGGGCUAGGGCUAACAGCA
hsa-miR-363-3p	AAUUGCACGGUAUCCAUCUGUA

hsa-miR-3613-5p	UGUUGUACUUUUUUUUUUGUUC
hsa-miR-130a-3p	CAGUGCAAUGUUAAAAGGGCAU
hsa-miR-10a-5p	UACCCUGUAGAUCCGAAUUUGUG
hsa-miR-32-5p	UAUUGCACAUUACUAAGUUGCA
hsa-miR-183-5p	UAUGGCACUGGUAGAAUUCACU
hsa-miR-17-5p	CAAAGUGCUUACAGUGCAGGUAG
hsa-miR-10b-5p	UACCCUGUAGAACCGAAUUUGUG
hsa-miR-20b-5p	CAAAGUGCUCAUAGUGCAGGUAG
hsa-miR-152-3p	UCAGUGCAUGACAGAACUUGG
hsa-let-7d-3p	CUAUACGACCUGCUGCCUUUCU
hsa-miR-140-5p	CAGUGGUUUUACCCUAUGGUAG
hsa-miR-424-5p	CAGCAGCAAUUCAUGUUUUGAA
hsa-miR-625-3p	GACUAUAGAACUUUCCCCCUCA
hsa-miR-514a-5p	UACUCUGGAGAGUGACAAUCAUG
hsa-miR-484	UCAGGCUCAGUCCCCUCCCGAU
hsa-miR-660-5p	UACCCAUUGCAUAUCGGAGUUG
hsa-miR-629-5p	UGGGUUUACGUUGGGAGAACU
hsa-miR-382-5p	GAAGUUGUUCGUGGUGGAUUCG
hsa-miR-199a-5p	CCCAGUGUUCAGACUACCUGUUC
hsa-miR-338-3p	UCCAGCAUCAGUGAUUUUGUUG
hsa-miR-224-5p	CAAGUCACUAGUGGUUCCGUU
hsa-miR-141-3p	UAACACUGUCUGGUAAAGAUGG
hsa-miR-320b	AAAAGCUGGGUUGAGAGGGCAA
hsa-miR-222-3p	AGCUACAUCUGGCUACUGGGU
hsa-miR-361-3p	UCCCCCAGGUGUGAUUCUGAUUU
hsa-miR-30e-3p	CUUUCAGUCGGAUGUUUACAGC
hsa-miR-423-3p	AGCUCGGUCUGAGGCCCCUCAGU
hsa-miR-151b/151a-5p	UCGAGGAGCUCACAGUCU
hsa-miR-106b-3p	CCGCACUGUGGGUACUUGCUGC
hsa-miR-99b-5p	CACCCGUAGAACCGACCUUGCG
hsa-miR-483-5p	AAGACGGGAGGAAAGAAGGGAG
hsa-miR-328-3p	CUGGCCCUCUCUGCCCUUCCGU

hsa-miR-3615	UCUCUCGGCUCCUCGCGGCUC
hsa-miR-1307-3p	ACUCGGCGUGGCGUCGGUCGUG
hsa-miR-301a-3p	CAGUGCAAUAGUAUUGUCAAAGC
hsa-miR-378a-3p	ACUGGACUUGGAGUCAGAAGGC
hsa-miR-197-3p	UUCACCACCUUCUCCACCCAGC
hsa-miR-215-5p	AUGACCUAUGAAUUGACAGAC
hsa-miR-4433b-5p	AUGUCCCACCCCCACUCCUGU
hsa-miR-320c	AAAAGCUGGGUUGAGAGGGU
hsa-miR-532-5p	CAUGCCUUGAGUGUAGGACCGU
hsa-miR-425-3p	AUCGGGAAUGUCGUGUCCGCCC
hsa-miR-145-5p	GUCCAGUUUUCCCAGGAAUCCCU
hsa-miR-1-3p	UGGAAUGUAAAGAAGUAUGUAU
hsa-miR-193a-5p	UGGGUCUUUGCGGGCGAGAUGA
hsa-miR-30b-5p	UGUAAACAUCCUACACUCAGCU
hsa-miR-15b-3p	CGAAUCAUUAUUUGCUGCUCUA
hsa-miR-590-3p	UAAUUUUAUGUAUAAGCUAGU
hsa-miR-18a-5p	UAAGGUGCAUCUAGUGCAGAUAG
hsa-miR-34a-5p	UGGCAGUGUCUUAGCUGGUUGU
hsa-miR-421	AUCAACAGACAUUAAUUGGGCGC
hsa-miR-130b-3p	CAGUGCAAUGAUGAAAGGGCAU
hsa-miR-196b-5p	UAGGUAGUUUCCUGUUGUUGGG
hsa-miR-106b-5p	UAAAGUGCUGACAGUGCAGAU
hsa-miR-181b-5p	AACAUUCAUUGCUGUCGGUGGGU
hsa-miR-625-5p	AGGGGGAAAGUUCUAUAGUCC
hsa-miR-324-5p	CGCAUCCCCUAGGGCAUUGGUGU
hsa-miR-542-3p	UGUGACAGAUUGAUAACUGAAA
hsa-miR-628-3p	UCUAGUAAGAGUGGCAGUCGA
hsa-miR-134-5p	UGUGACUGGUUGACCAGAGGGG
hsa-miR-3152-5p	AUUGCCUCUGUUCUAACACAAG
hsa-miR-374a-3p	CUUAUCAGAUUGUAUUGUAAUU
hsa-miR-206	UGGAAUGUAAGGAAGUGUGUGG
hsa-miR-200a-3p	UAACACUGUCUGGUAACGAUGU

hsa-miR-199b-5p	CCCAGUGUUUAGACUAUCUGUUC
hsa-miR-203a-3p	GUGAAAUGUUUAGGACCACUAG
hsa-miR-942-5p	UCUUCUCUGUUUUGGCCAUGUG
hsa-miR-4732-5p	UGUAGAGCAGGGAGCAGGAAGCU
hsa-miR-1306-5p	CCACCUCCCCUGCAAACGUCCA
hsa-miR-574-3p	CACGCUCAUGCACACACCCACA
hsa-miR-370-3p	GCCUGCUGGGGUGGAACCUGGU
hsa-miR-503-5p	UAGCAGCGGGAACAGUUCUGCAG
hsa-miR-6126	GUGAAGGCCCGGCGGAGA
hsa-miR-339-3p	UGAGCGCCUCGACGACAGAGCCG
hsa-miR-664a-5p	ACUGGCUAGGGAAAAUGAUUGGAU
hsa-miR-92b-3p	UAUUGCACUCGUCCCGGCCUCC
hsa-miR-1301-3p	UUGCAGCUGCCUGGGAGUGACUUC
hsa-miR-4732-3p	GCCCUGACCUGUCCUGUUCUG
hsa-miR-374b-5p	AUAUAAUACAACCUGCUAAGUG
hsa-miR-4730	CUGGCGGAGCCCAUUCCAUGCCA
hsa-miR-1277-3p	UACGUAGAUAUAUAUGUAUUUU
hsa-miR-598-3p	UACGUCAUCGUUGUCAUCGUCA
hsa-miR-3666	CAGUGCAAGUGUAGAUGCCGA
hsa-miR-95-3p	UUCAACGGGUAUUUAUUGAGCA
hsa-miR-3915	UUGAGGAAAAGAUGGUCUUAUU
hsa-miR-139-5p	UCUACAGUGCACGUGUCUCCAGU
hsa-miR-200c-3p	UAAUACUGCCGGGUAAUGAUGGA
hsa-miR-29c-5p	UGACCGAUUUCUCCUGGUGUUC
hsa-miR-652-3p	AAUGGCGCCACUAGGGUUGUG
